# PDGFRα and αSMA mark two distinct mesenchymal cell populations involved in parenchymal and vascular remodeling in pulmonary fibrosis

**DOI:** 10.1152/ajplung.00128.2019

**Published:** 2020-02-05

**Authors:** Valentina Biasin, Slaven Crnkovic, Anita Sahu-Osen, Anna Birnhuber, Elie El Agha, Katharina Sinn, Walter Klepetko, Andrea Olschewski, Saverio Bellusci, Leigh M. Marsh, Grazyna Kwapiszewska

**Affiliations:** ^1^Division of Endocrinology and Diabetology, Department of Internal Medicine, Medical University of Graz, Graz, Austria; ^2^Ludwig Boltzmann Institute for Lung Vascular Research, Graz, Austria; ^3^Otto Loewi Research Center, Division of Physiology, Medical University of Graz, Graz, Austria; ^4^Excellence Cluster Cardio-Pulmonary System (ECCPS), Member of the German Center for Lung Research (DZL), Universities of Giessen and Marburg Lung Center (UGMLC), Justus Liebig University Giessen, Giessen, Germany; ^5^Division of Thoracic Surgery, Department of Surgery, Medical University of Vienna, Austria; ^6^Experimental Anesthesiology, Department of Anesthesiology and Intensive Care Medicine, Medical University of Graz, Graz, Austria

**Keywords:** collagen, fibroblasts, fibrosis, myofibroblasts, transdifferentiation

## Abstract

Pulmonary fibrosis is characterized by pronounced collagen deposition and myofibroblast expansion, whose origin and plasticity remain elusive. We utilized a fate-mapping approach to investigate α-smooth muscle actin (αSMA)+ and platelet-derived growth factor receptor α (PDGFRα)+ cells in two lung fibrosis models, complemented by cell type-specific next-generation sequencing and investigations on human lungs. Our data revealed that αSMA+ and PDGFRα+ cells mark two distinct mesenchymal lineages with minimal transdifferentiation potential during lung fibrotic remodeling. Parenchymal and perivascular fibrotic regions were populated predominantly with PDGFRα+ cells expressing collagen, while αSMA+ cells in the parenchyma and vessel wall showed variable expression of collagen and the contractile protein desmin. The distinct gene expression profile found in normal conditions was retained during pathologic remodeling. Cumulatively, our findings identify αSMA+ and PDGFRα+ cells as two separate lineages with distinct gene expression profiles in adult lungs. This cellular heterogeneity suggests that anti-fibrotic therapy should target diverse cell populations.

## INTRODUCTION

Pulmonary fibrosis is characterized by progressive scarring and stiffening of the lung, which lead to significant functional impairment and ultimately death (18, 20). These processes are not only limited to the lung parenchyma but also affect the vascular bed. Vascular remodeling leads to lumen reduction and vessel occlusion (11), resulting in pulmonary hypertension (PH) and worsened patient survival (6, 13).

Remodeling in both the parenchymal and vascular compartments is characterized by aberrant cellular proliferation, enhanced extracellular matrix (ECM) deposition, and increased tissue stiffness. Expansion of α-smooth muscle actin (αSMA)-expressing cells, termed myofibroblasts, is thought to be the major pathomechanism responsible for vascular and parenchymal remodeling. While resident αSMA+ cells are indeed a major pathological cell type that expands and contributes to pulmonary vascular remodeling (7, 28), neither vascular nor airway smooth muscle cells represent the major source of interstitial myofibroblasts in lung parenchymal remodeling (10). Furthermore, the simplistic paradigm of myofibroblasts as the single most important cell type in lung fibrosis has been questioned by a recent fate-mapping approach, which showed that αSMA+ cells are not the main source of collagen production (29). After bleomycin-induced lung injury, not only myofibroblasts but also multiple resident cell populations expand and are involved in collagen production (27, 32).

During late lung development in mouse, collagen-I-expressing platelet-derived growth factor receptor α (PDGFRα)+ cells are the source of lung alveolar myofibroblasts (21). However, in adult animals there are strong compartmental differences in PDGFRα and αSMA expression. We have recently demonstrated that PDGFRα expression marks a population of αSMA negative perivascular (adventitial) fibroblasts (7). Furthermore, after bleomycin challenge, interstitial PDGFRα+ cells are αSMA negative, while perivascular and peribronchial PDGFRα+ cells express variable amounts of αSMA (27). Cumulatively, these studies indicate that αSMA+ and PDGFRα+ cells might represent two major pathogenic mesenchymal populations, possessing distinct compartment-specific responses and behavior during fibrotic remodeling in the adult lung. As such, it is still unclear whether αSMA+ cells in particular lung niches are derived from PDGFRα+ cells and whether αSMA+ cells are the sole contributor to collagen production in lung fibrosis.

In the current study, we investigated the cellular fates of PDGFRα+ and αSMA+ cells in two animal models displaying lung parenchymal and vascular remodeling with associated PH. Our genetic labeling strategy combined with immunofluorescence allowed investigating transdifferentiation events between the two populations. The contribution of both cell types was anatomically annotated and divided into parenchymal and vascular compartments in lung tissue samples from both animal models as well as human donor and end-stage idiopathic pulmonary fibrosis patients. This comprehensive methodological approach revealed distinct fates of PDGFRα+ and αSMA+ cells, their association with collagen production, and a compartment-specific contribution to lung fibrosis.

## MATERIALS AND METHODS

Full experimental details are given in the online supplement at the following link: https://doi.org/10.5281/zenodo.3532795.

### Human Lung Tissue

Lung tissue from idiopathic pulmonary fibrosis (IPF) patients undergoing lung transplantation were obtained from the Department of Surgery, Division of Thoracic Surgery, Medical University of Vienna, Austria, following written informed consent and approval by the institutional ethics committee (976/2010). Samples of downsized transplant donor lungs served as controls.

### Experimental Animals

Mice (C57BL6 background) carrying tamoxifen-responsive Cre recombinase under the *Pdgfra* (*Tg(Pdgfra-cre/ERT2)1Wdr*) and *Acta2* (*Tg(Acta2-cre/ERT2)12Pcn*) promoters (26, 31) were crossed with *tdTomato^flox^* (*Gt(ROSA)26Sortm14(CAG-tdTomato)Hze/J*) reporter mice (Jackson Laboratory, Bar Harbor, ME). Mice (C57BL6/CBA background) carrying ectopic overexpression of the Ap-1 transcription factor member *Fosl2* (*Tg(H2-K-Fosl2^EGFP^) 13Wag*) (9) were crossed with *Pdgfra-* or *Acta2-CreERT2; tdTomato^flox^* reporter mice. All animal experiments were approved by the local authorities (Austrian Ministry of Education, Science and Culture) and performed in accordance with the EU directive 2010/63/EU.

### Tamoxifen and Bleomycin Administration

Male and female mice were used for control/bleomycin and littermates/Fra-2 tg mice respectively. Tamoxifen was given via food 5 days after bleomycin or saline injection in *Acta2-CreERT2; tdTomato^flox^* mice to label myofibroblasts during fibrosis development [as previously published (10)] and as single daily ip injections (3 mg in 90% corn oil, 10% ethanol; Sigma Aldrich, Vienna, Austria) for 3–5 consecutive days in bleomycin and control *Pdgfra-CreERT2; tdTomatoflox* mice, in 7- to 8-wk-old *Fosl2tg; Acta2-CreERT2; tdTomatoflox*, in *Fosl2tg; Pdgfra-CreERT2; tdTomatoflox* mice, and littermate controls (Supplemental Fig. S1; https://doi.org/10.5281/zenodo.3532795). Bleomycin (2 U/kg body weight) (Sigma Aldrich, Vienna, Austria) or saline was applied intratracheally with a MicroSprayer Aerosolizer (Penn-Century, Wyndmoor, PA) under light (~2%) isoflurane anesthesia. Lungs were harvested 2 wk after bleomycin instillation (2). *Fosl2tg; Pdgfra-CreERT2; tdTomatoflox, Fosl2tg; Acta2-CreERT2; tdTomatoflox*, and littermate controls were euthanized, and lungs harvested at 20 wk of age.

### Immunofluorescence Staining and Analysis

Mouse lungs were perfused with PBS followed by inflation with optimal cutting temperature compound (Tissue Tek, Sakura, CA), fixed overnight in 1% paraformaldehyde followed by overnight dehydration in 30% sucrose and stored at −80°C. Cryo-sections were treated with ice-cold methanol/acetone, blocked with 2.5% horse serum (Vector Laboratories, Palo Alto, CA), and stained with antibodies against PDGFRα (1:1,000; Cell Signaling, Leiden, The Netherlands), αSMA-Cy3 (1:200, Sigma Aldrich), αSMA-FITC (1:100, Sigma Aldrich), desmin (1:100, R&D systems, Minneapolis MN), pro-collagen I (1:500, Southern Biotech, Birmingham AL), and von Willebrand factor (vWF) (1:100; Dako/Agilent, Santa Clara, CA). Sections were incubated with respective secondary antibodies conjugated with Alexa Fluor 488, 555, or 647 (1:500, all from ThermoFisher Scientific, Bonn, Germany) and mounted with DAPI containing mounting media (Vector Laboratories). PDGFRα signal was enhanced by the Tyramide Signal Amplification Kit (TSA-488, ThermoFisher Scientific) according to the manufacturer’s instruction. For each sample 10–15 images from random areas were acquired at ×40 magnification (Image size x: 225 µm; y: 22 5µm) under an LSM510 confocal microscope (Zeiss, Oberkochen, Germany). Vascular compartment was identified by characteristic morphological appearance (Supplemental Fig. S2; https://doi.org/10.5281/zenodo.3532795) and validated by vWF endothelial staining. Parenchymal regions were defined by absence of vascular structure and vWF staining within the imaged region. Manual counting of tdTomato-, AF488-, and AF647-positive cells was performed by two independent investigators from 3–10 *XY* panels from each sample. Lineage-traced cells containing obvious nuclei were counted. Brightfield picture image was merged to the immunofluorescence signal to help delineating the cell border; however, in unclear cases the signal/cells were not included in the analysis. Blinding of samples was not possible due to obvious morphological differences in the lung tissue.

### RNA Sequencing

The left lung lobe from tdTomato mice was mechanically separated with two scalpels followed by incubation with dispase (50 U/mL, Corning, NY) for 1 h at 37°C to generate a single cell suspension. tdTomato-positive cells were sorted directly into RNA lysis buffer (Qiagen, Venlo, The Netherlands) using a FACSAria II cell sorter (BD Biosciences, San Jose, CA). RNA was isolated with the RNeasy micro kit (Qiagen). Library preparation using the SmartSeq2 protocol and sequencing on the Illumina HiSeq 3000/4000 platform was done by the Biomedical Sequencing Facility (CeMM, Vienna, Austria). Next-generation sequencing reads were aligned with the TopHat2 (v2.1.1) (12) splice junction mapper using the Bowtie2 short read aligner (v2.2.9) (16). Transcriptome assembly and differential expressing calling was performed with Cufflinks (v2.1.1) (30). A detailed analysis of the initial analysis can be found in the online supplement. Differentially regulated genes were ranked according to their log-fold-change and their significance (q-value). Prcomp was used to calculate the principal components; the first two principal components were plotted using the ggplot2 package. For generation of heatmaps, data were transformed to log2(FPKM+1). Gene enrichment analysis was performed using EnrichR (5, 15). The data discussed in this publication have been deposited in the National Center for Biotechnology Information's Gene Expression Omnibus (GEO) (8) and are accessible through GEO Series accession number GSE126205.

### Public Data Set Analysis

#### Human.

scRNA-Seq data from Reyfman et al. (25) was downloaded from GEO (GSE122960), and the raw count matrices in HDF5 format imported and analyzed in Seurat 3.1.2 (https://linkinghub.elsevier.com/retrieve/pii/S0092867419305598). Four donor samples (GSM3489182, GSM3489185, GSM3489187, GSM3489189) and four IPF samples (GSM3489183, GSM3489184, GSM3489188, GSM3489190) were individually processed and normalized using the SCTransform (10a) function removing cells with >10% mitochondrial percentage.Samples were concatenated using SCTransform, and dimension reduction was performed by PCA and t-SNE using default parameters. Cells were clustered at a resolution of 0.4. Fibroblasts clusters were identified by fibroblast markers as identified in Ref. 25.

#### Mouse.

scRNA-Seq data from Peyser et al. (23) was downloaded from GEO (GSE129605), and the Feature-Barcode Matrices were imported in and analyzed in Seurat. Samples were individually processed, removing cells with high mitochondrial percentage >5%, and data were normalized using default parameters. Samples were concatenated using a precomputed anchor set identified by the function FindIntegrationAnchors. Concatenated samples were then scaled to regress out differences in number of features per cell, and dimension reduction was performed by PCA and t-SNE using default parameters. Clustering was performed at a resolution of 0.3. Fibroblast clusters were annotated by cluster alignment against collected mouse data sets available at GEO (1) using SingleR.

### Statistical Analysis

Statistical analysis was performed with GraphPad Prism 5 and bioinformatic analysis was performed with RStudio (https://www.rstudio.com) and R (www.r-project.org) (version number 3.4.1). Data are presented as mean with SD in all graphs. Statistical differences between the groups were determined by using two-way ANOVA with Bonferroni post hoc comparison test. *P* values <0.05 were considered significant.

## RESULTS

### αSMA and PDGFRα-expressing Cells Represent Distinct Subsets of Collagen-producing Fibroblasts in Human Lungs

**Fig. 1.**
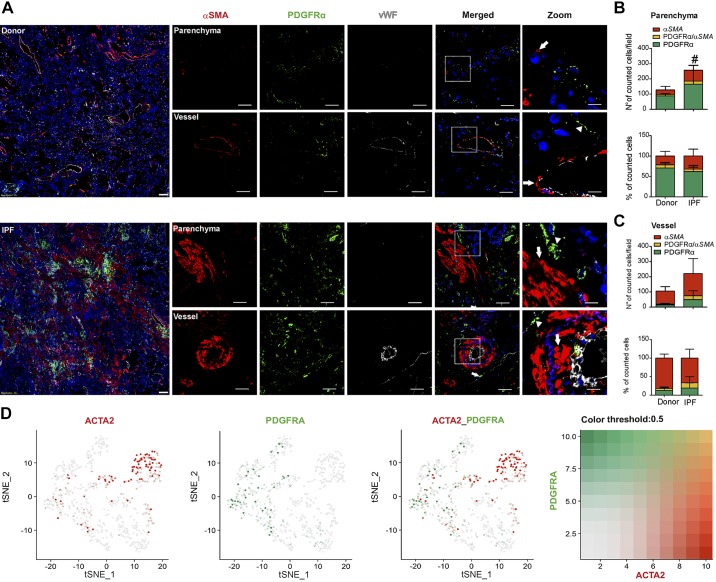
α-Smooth muscle actin (αSMA)- and platelet-derived growth factor receptor-α (PDGFRα)-expressing cells represent two distinct populations of fibroblasts in human idiopathic pulmonary fibrosis (IPF) lungs. *A*: immunofluorescence staining of human donor and IPF sections for αSMA, PDGFRα, and von Willebrand factor (vWF). Parenchymal and vascular regions are shown. Scale bar: overview 200 µm; single staining and merged 50 µm; zoomed merged 12.5 µm. Arrows: single αSMA-positive cells; arrowheads: single PDGFRα-positive cells; asterisk: double αSMA/PDGFRα-positive cells. Number and percentage of positive cells for single αSMA, single PDGFRα, and double αSMA/PDGFRα-positive cells in the parenchyma (*B*) and in the vasculature (*C*). Cell counts were performed on 3–5 images for each donor and IPF section from *n* = 6–8 different donors and IPF lungs. *P* < 0.05 was considered significant. #Differences between single PDGFRα-positive cells in IPF compared with donor lungs. *D*: *t*-distributed stochastic neighbor embedding (tSNE) plots showing expression of ACTA2 and PDGFRA expression on the fibroblasts cluster. Data from Reyfman et al. data set (GSE122960) (25).

To determine the relative contribution of αSMA and PDGFRα cells in parenchymal and vascular remodeling associated with lung fibrosis, multicolor immunofluorescent staining against PDGFRα, αSMA, and vWF was performed. Increased numbers of both αSMA+ (~2.5-fold) and PDGFRα+ (~2-fold) cells were observed in the lung parenchyma of patients with IPF compared with donors (Fig. 1, *A* and *B*). The mean percentage of myofibroblasts (αSMA+ cells) in the fibroblast cell pool of the parenchymal compartment (total number of αSMA+ and PDGFRα+ cells) was increased from ~20% in the donors to ~30% of the IPF patients (Fig. 1*B*). The vast majority of cells were positive for either αSMA or PDGFRα, while the percentage of double αSMA+/PDGFRα+ cells reached maximally 12% in IPF lungs (Fig. 1*B*).

In the vascular compartment of IPF patients, we observed an increase in both PDGFRα+ (~3-fold) and αSMA+ (~2-fold) cells (Fig. 1*C*). The percentage of double positive cells was low in donors (~5%) and increased (to ~15%) in IPF remodeled vessels (Fig. 1*C*). The localization pattern was unchanged between donor and IPF vasculature; in both groups αSMA+ cells were confined to vascular regions up to the external elastic lamina (Supplemental Fig. S2, dotted line; https://doi.org/10.5281/zenodo.3532795), while PDGFRα+ cells were present almost exclusively in perivascular regions (Fig. 1*A*, zoomed area, Supplemental Fig. S2, continuous line; https://doi.org/10.5281/zenodo.3532795). Importantly, the investigation of an independent scRNA-Seq data set (GSE122960) analyzing human IPF and donor lungs (25) revealed that, within the fibroblast cluster, ACTA2- and PDGFRA-expressing cells delineate two distinct subclusters with minimal overlap (Fig. 1*D*). This finding corroborates and reinforces our observations.

**Fig. 2.**
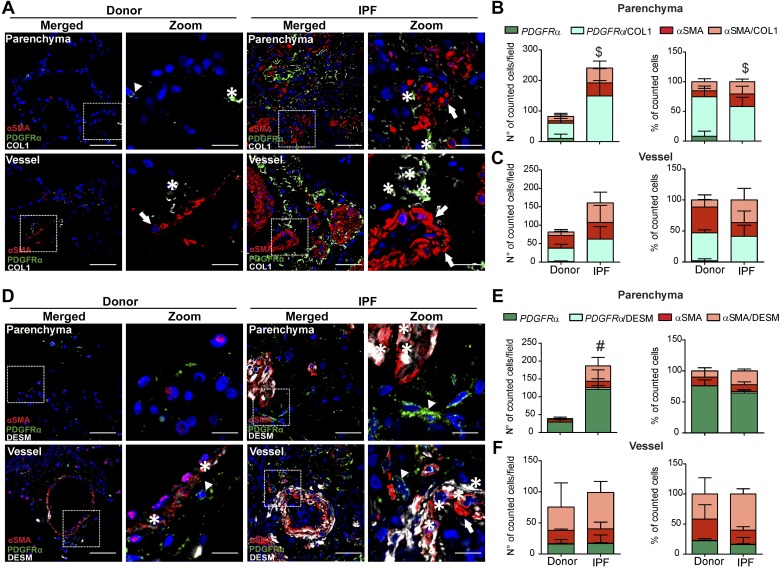
Both α-smooth muscle actin (αSMA)- and platelet-derived growth factor receptor-α (PDGFRα)-expressing cells contribute to collagen production, but desmin (DES) is expressed mostly by αSMA-expressing cells in human idiopathic pulmonary fibrosis (IPF) lungs. *A*: multicolor immunofluorescence staining of human donor and IPF sections for αSMA, PDGFRα, and collagen 1 (COL1). Parenchymal and vascular regions are shown. Scale bar: merged 50 µm; zoomed 12.5 µm. Arrows: single αSMA-positive cells; arrowheads: single PDGFRα-positive cells; asterisk: double αSMA/COL1 or PDGFRα/COL1-positive cells. Number and percentage of single αSMA, single PDGFRα, double αSMA /COL1, and double PDGFRα /COL1-positive cells in the parenchyma (*B*) and in the vasculature (*C*). Cell count was performed on 3–6 images for each donor and IPF section from *n* = 3–4 different donors and IPF lungs. *P* value < 0.05 was considered significant. $Differences between double PDGFRα/COL1-positive cells in IPF compared with donor lungs. *D*: multicolor immunofluorescence staining of human donor and IPF sections for αSMA, PDGFRα, and DES. Parenchymal and vascular region are shown. Scale bar: merged 50 µm; zoomed merged 12.5 µm. Arrows: single αSMA-positive cells; arrowheads: single PDGFRα-positive cells; asterisk: double αSMA/DES or PDGFRα/DES-positive cells. Number and percentage of single αSMA, single PDGFRα, double αSMA/DES, and double PDGFRα/DES-positive cells in the parenchyma (*E*) and in the vasculature (*F*). Cell count was performed in 3–6 images for each donor and IPF section from *n* = 2–3 different donors and IPF. *P* value < 0.05 was considered significant. #Differences between single PDGFRα-positive cells in IPF compared with donor lungs.

We next assessed the contribution of αSMA+ and PDGFRα+ cells to collagen production. In both compartments of donor and IPF patients, approximately half of αSMA+ cells displayed intracellular costaining with collagen (COL1), while almost all PDGFRα+ cells were COL1 positive (Fig. 2, *A*–*C*). Preferential expression of collagens or contractile components is traditionally used to further subclassify αSMA+ cells into synthetic or contractile phenotype (24). We therefore addressed the expression of the contractile marker desmin (DES) in αSMA+ and PDGFRα+ cells. As shown in Fig. 2, *D*–*F*, DES almost exclusively colocalized with αSMA+ cells. In IPF lungs the percentage of αSMA+/DES+ cells increased compared with donors in both parenchymal and vascular compartment (Fig. 2, *E* and *F*).

### αSMA+ Cells Are a Separate Subpopulation of Fibroblasts with Compartment-specific Contribution to Remodeling

Our human data revealing limited overlap between αSMA+ and PDGFRα+ cells, including their divergent expression of collagen and desmin, indicated that these cell populations might represent two main and distinct fibroblast populations in the adult lung. We therefore employed a fate-mapping approach using transgenic mice to assess lineage hierarchy and possible transdifferentiation between αSMA- and PDGFRα-expressing cells. We employed two murine models in which the parenchymal and vascular remodeling process is driven by two different underlying pathomechanisms: *1*) bleomycin-induced lung fibrosis and *2*) ectopic overexpression of *Fra-2* (*Fra-2 Tg*). Both models accumulate αSMA+ cells and collagen in parenchymal regions (Supplemental Fig. S3, *A–D*; https://doi.org/10.5281/zenodo.3532795), have increased coverage of distal pulmonary vessels by αSMA+ cells with deposition of intra-/perivascular collagen (Supplemental Fig. S4, *A–D*; https://doi.org/10.5281/zenodo.3532795), and have impaired lung function with restrictive phenotype (2–4).

Resident and newly formed αSMA+ cells in bleomycin-induced fibrosis were genetically labeled using the *Acta2-CreERT2; tdTomato^flox^* mouse line (*Acta2-tdT*) as previously described (10), and cellular changes were investigated in the lung 2 wk after bleomycin application (Supplemental Fig. S1; https://doi.org/10.5281/zenodo.3532795).

**Fig. 3.**
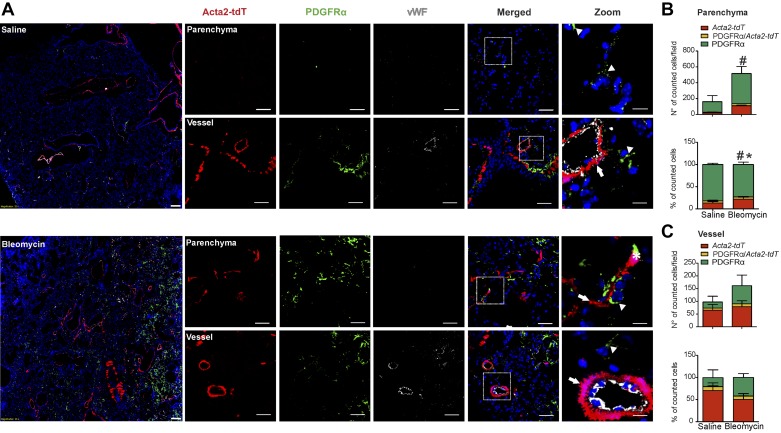
Lineage-traced α-smooth muscle actin (αSMA)-expressing cells contribute to parenchymal and vascular remodeling in bleomycin model. *A*: lineage tracing of αSMA-positive cells (*Acta2-tdT*) and immunofluorescence staining for platelet-derived growth factor receptor-α (PDGFRα) and von Willebrand factor (vWF) was performed in saline- and bleomycin-treated mice. Parenchymal and vascular regions are shown. Scale bar: overview 200 µm; single staining and merged 50 µm; zoomed merged 12.5 µm. Arrows: single *Acta-tdT*-positive cells; arrowheads: single PDGFRα-positive cells; asterisk: double *Acta-tdT*/PDGFRα-positive cells. Number and percentage of *Acta2-tdT* and PDGFRα-positive cells in the parenchyma (*B*) and in the vasculature (*C*). Cell count was performed in 3–8 images for each mouse section from *n* = 3–4 different saline- and bleomycin-treated mice. *P* value < 0.05 was considered significant. **#**Differences between single PDGFRα-positive cells; *Differences between single *Acta2-tdT* positive cells in bleomycin- compared with saline-treated mice.

In the parenchymal compartment of bleomycin-treated mice, the total number of *Acta2-tdT* cells increased (~5-fold) compared with the saline control group (Fig. 3, *A* and *B*). However, additional immunostaining against PDGFRα revealed that the vast majority of cells in fibrotic regions were PDGFRα+ and that *Acta2-tdT* cells only represented ~20% of the total fibroblast pool (total number of αSMA+ and PDGFRα+ cells) (Fig. 3, *A* and *B*). In contrast, the vascular compartment contained mostly lineage-labeled *Acta2-tdT* cells (Fig. 3*C*). Postbleomycin, despite a strong increase in number of PDGFRα+ cells, the relative proportion of both cell types remained relatively constant in the parenchyma (Fig. 3*B*), suggesting an equal expansion of both cell population in bleomycin. In contrast, in the vasculature, the percentage of PDGFRα+ cells increased compared with the percentage of *Acta-tdT*+ cells (Fig. 3*C*). In both saline- and bleomycin-treated mice only a limited percentage of cells (~5–8%) in either compartment coexpressed both markers (Fig. 3, *B* and *C*).

**Fig. 4.**
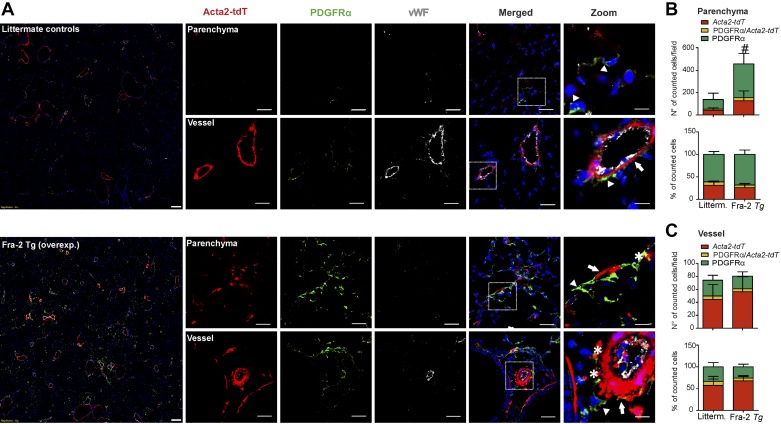
Lineage-traced α-smooth muscle actin (αSMA)-expressing cells contribute to parenchymal and vascular remodeling in *Fra-2 Tg* mice. *A*: lineage tracing of αSMA-positive cells (*Acta2-tdT*) and immunofluorescence staining for platelet-derived growth factor receptor-α (PDGFRα) and von Willebrand factor (vWF) was performed in wild-type (wt) littermates and *Fra-2 tg* mice. Parenchymal and vascular regions are shown. Scale bar: overview 200 µm; single staining and merged 50 µm; zoomed merged 12.5 µm. Arrows: single *Acta2-tdT*-positive cells; arrowheads: single PDGFRα-positive cells; asterisk: double *Acta2-tdT* /PDGFRα-positive cells. Number and percentage of single *Acta2-tdT*, single PDGFRα and *Acta2-tdT*/PDGFRα double positive cells in the parenchyma (*B*) and in the vasculature (*C*). Cell count was performed in 4–9 images for each mouse section from *n* = 3–5 different wt littermate controls and *Fra-2 Tg* mice. *P* value < 0.05 was considered significant. **#**Differences between single PDGFRα-positive cells in *Fra-2 Tg* mice compared with wt littermates.

Observations from the bleomycin model were compared with an alternative pulmonary fibrosis model (1a, 3). *Fra-2 Tg* mice develop progressive and irreversible parenchymal and vascular remodeling (Supplemental Figs. S3 and S4; https://doi.org/10.5281/zenodo.3532795) (1a, 3). Remodeling is first observable only in the vascular compartment and accompanied by PH at the age of 7–8 wk (1a). Over the course of the next few weeks, parenchymal changes are visible, starting first with perivascular and interstitial inflammation at 12–16 wk and progressing to fibrosis-characteristic restrictive lung function impairment (3, 4). Genetic labeling of Acta2-expressing cells was performed in *Fra-2 Tg* mice carrying the *Acta2-CreERT2; tdTomato^flox^* alleles before parenchymal changes and cell fate were investigated at 20 wk (Supplemental Fig. S1; https://doi.org/10.5281/zenodo.3532795). In the parenchymal regions of Fra-2 *Tg* mice, we observed an increase in *Acta2-tdT* cells (~4.5-fold) compared with littermate control mice (Fig. 4, *A* and *B*). In *Fra-2 Tg* mice, *Acta2-tdT* cells constituted a higher proportion of cells than in the bleomycin model (~30% vs. ~20%, respectively; Fig. 3, *B* and *C*). In the vasculature of *Fra-2 Tg* mice, the number of *Acta2-tdT* cells was increased (~1.5-fold) (Fig. 4*C*) compared with littermate control mice, which is consistent with more pronounced vascular remodeling in *Fra-2 Tg* mice (Supplemental Fig. S4, *B* and *D*; https://doi.org/10.5281/zenodo.3532795). The relative proportion of *Acta2-tdT* and PDGFRα+ cells was unaltered in the parenchymal and vascular compartments of both *Fra-2 Tg* and control mice (Fig. 4, *B* and *C*). Similar to the bleomycin model, double *Acta2-tdT*/PDGFRα+ cells represented a minor subpopulation (~5–8%) in either compartment (Fig. 4, *B* and *C*).

### PDGFRα-expressing Cells without αSMA Expression Constitute the Bulk of Cells in Parenchymal Remodeling

**Fig. 5.**
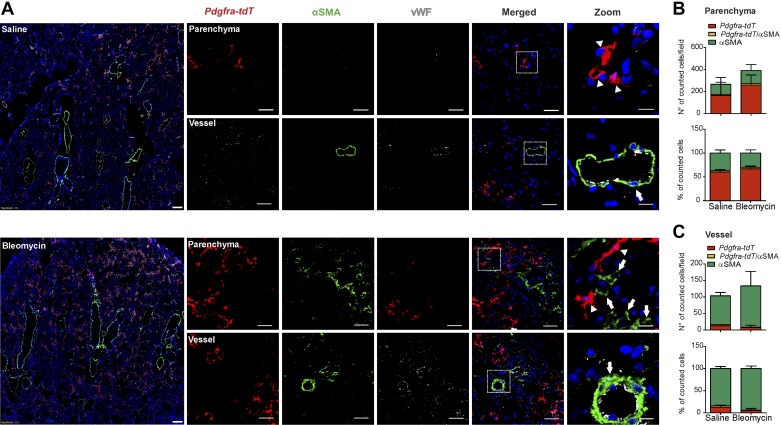
Lineage-traced platelet-derived growth factor receptor-α (PDGFRα)-positive cells strongly contribute to parenchymal remodeling in bleomycin model. *A*: lineage tracing of PDGFRα-positive cells (*Pdgfra-tdT*) and immunofluorescence staining for α-smooth muscle actin (αSMA) and von Willebrand factor (vWF) was performed in saline- and bleomycin-treated mice. Parenchymal and vascular regions are shown. Scale bar: overview 200 µm; single staining and merged 50 µm; zoomed merged 12.5 µm. Arrows: single αSMA-positive cells; arrowheads: single *Pdgfra-tdT*-positive cells; asterisk: double αSMA/ *Pdgfra-tdT*-positive cells. Number and percentage of single αSMA, single *Pdgfra-tdT*, and αSMA/*Pdgfra-tdT* double positive cells in the parenchyma (*B*) and in the vasculature (*C*). Cell count was performed in 6–10 images for each mouse section from *n* = 3–5 different saline- and bleomycin-treated mice. *P* value < 0.05 was considered significant.

**Fig. 6.**
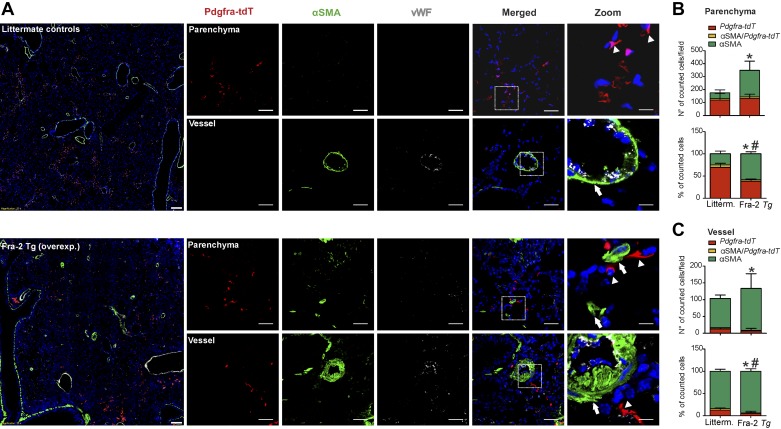
Lineage-traced platelet-derived growth factor receptor-α (PDGFRα)-positive cells partially contribute to parenchymal remodeling in Fra-2 Tg mice. Lineage tracing of PDGFRα-positive cells (*Pdgfra-tdT*) and immunofluorescence staining for α-smooth muscle actin (αSMA) and von Willebrand factor (vWF) was performed in wild-type (wt) littermates and *Fra-2 Tg* mice *A*: parenchymal and vascular regions are shown. Scale bar: overview 200 µm; single staining and merged 50 µm; zoomed merged 12.5 µm. Arrows: single αSMA-positive cells; arrowheads: single *Pdgfra-tdT*-positive cells; asterisk: double αSMA/*Pdgfra-tdT*-positive cells. Number and percentage of single αSMA, single *Pdgfra-tdT*, and αSMA/*Pdgfra-tdT* double positive cells in the parenchyma (*B*) and in the vasculature (*C*). Cell count was performed in 6–10 images for each mouse section from *n* = 4 different littermate controls and *Fra-2 Tg mice*. *P* value < 0.05 was considered significant. #Differences between single *Pdgfra-tdT*-positive cells; *differences between single αSMA-positive cells in *Fra-2 Tg* mice compared with wt littermates.

The low percentage of double *Acta2-tdT*/PDGFRα+ cells prompted us to validate these results by a reciprocal approach. Here the fate of PDGFRα+ cells was followed using a second lineage-tracing tool, *Pdgfra-CreERT2; tdTomato^flox^* (*Pdgfra-tdT*) in the bleomycin and *Fra-2 Tg* models (Figs. 5 and 6). The colocalization of cytoplasmic tdTomato reporter and plasma membrane PDGFRα was considered positive only if both signals were present in the same cell (Supplemental Fig. S1, dashed lines and arrows; https://doi.org/10.5281/zenodo.3532795).

An expansion of *Pdgfra-tdT* cells was observed in the parenchymal regions of bleomycin compared with saline-treated mice (~1.5-fold increase; Fig. 5, *A* and *B*). No change in the percentage of double *Pdgfra-tdT*/αSMA+ cells was observed in the parenchyma of bleomycin-treated mice (~3%, Fig. 5*B*). In line with the PDGFRα immunostaining of the *Acta2-tdT* line (Fig. 4), lineage-labeled *Pdgfra-tdT* cells represented the majority of cells in fibrotic regions and only a minority in the vascular compartment of bleomycin mouse model (Fig. 5*C*). *Pdgfra-tdT* cells were mostly confined to the perivascular region and accounted for ~12% of cells in bleomycin-treated mice (Fig. 5*C*). Double *Pdgfra-tdT*/αSMA+ cells were not detected in the vessels of saline mice, while in bleomycin-treated mice their percentage increased to 4% (Fig. 5*C*).

*Fra-2 Tg* mice displayed similar number of *Pdgfra-tdT* cells compared with littermate control mice (Fig. 6, *A* and *B*), but the resulting ratio of *Pdgfra-tdT* to αSMA+ cells decreased in the lung parenchyma, changing from 70% in controls to 40% in *Fra-2 Tg* mice (Fig. 6*B*). Again, the presence of *Pdgfra-tdT*/αSMA double positive cells was a rare event (~3–5%) in *Fra-2 Tg* and littermate controls (Fig. 6*B*). In the lung vasculature the number of perivascular *Pdgfra-tdT* cells did not change significantly in *Fra-2 Tg* compared with control littermates (Fig. 6*C*). Again, we observed almost no double *Pdgfra-tdT*/αSMA+ cells in the vascular compartment of normal and remodeled vessels (Fig. 6*C*).

### αSMA and PDGFRα Cells Are Characterized by a Separate and Distinct Gene Expression Profile

**Fig. 7.**
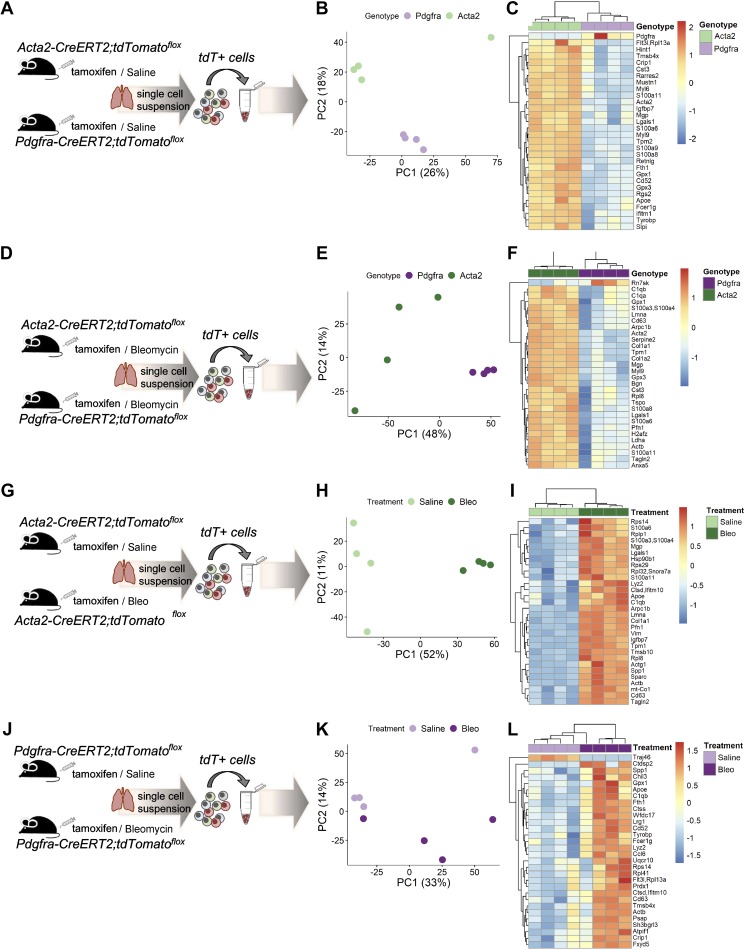
α-Smooth muscle actin (αSMA)- and platelet-derived growth factor receptor-α (PDGFRα)-expressing cells present a distinct gene expression. *A, D, G, J*: schematic representation of the applied workflow for RNA sequencing. *B, E, H, K*: principal component analysis (PCA) of the normalized RNA-Seq data transcripts of the reported groups. *C, F, I, L*: heatmap of hierarchical clustering showing the top 30 differentially expressed protein-coding genes between the reported groups.

Both our human data and lineage tracing experiments indicated that αSMA- and PDGFRα-expressing cells represent two largely separate populations in the normal and fibrotic adult lungs with apparently limited transdifferentiation capacity. We next expanded our investigations and performed RNA sequencing on tdTomato-positive cells sorted from the lungs of *Acta2-tdT* and *Pdgfra-tdT* mice (Fig. 7). Representative examples of gating strategy for *Acta2-tdT* and *Pdgfra-tdT* cells are shown in Supplemental Fig. S5 (https://doi.org/10.5281/zenodo.3532795). Principal component analysis (PCA) of the global gene expression profiles and heatmap analysis revealed that *Acta2-tdT* and *Pdgfra-tdT* cells indeed represent two distinct cellular clusters in normal adult murine lungs (Fig. 7, *B* and *C*). Gene ontology (GO) analysis of molecular functions revealed that *Acta2-tdT* cells were enriched in actin and tropomyosin binding, suggesting a predominance of the contractile phenotype in these cells, while *Pdgfra-tdT* cells in MAPK kinase binding and complement component and PDGF binding (Supplemental Table S1*A*; https://doi.org/10.5281/zenodo.3532795). Although bleomycin challenge (Fig. 7*D*) induced significant gene expression changes in both *Acta2-tdT* and *Pdgfra-tdT* cell populations, it did not affect *Acta2-tdT* and *Pdgfra-tdT* distinct cellular clusters (Fig. 7, *E* and *F*, and Supplemental Table S1B; https://doi.org/10.5281/zenodo.3532795). As expected, bleomycin induced significant changes in both *Acta2-tdT* and *Pdgfra-tdT* cells compared with saline treatment (Fig. 7, *G*–*I* and *J*–*L*). Our GO molecular functions were unique for each population. RNA binding, translation factor activity, and integrin binding were among the 20 most enriched molecular functions in *Acta2-tdT* cells from bleomycin-treated mice (Supplemental Table S1C; https://doi.org/10.5281/zenodo.3532795). In the *Pdgfra-tdT* population, there was enrichment for endopeptidase activity and cholesterol transporter activity upon bleomycin treatment (Supplemental Table S1*C*; https://doi.org/10.5281/zenodo.3532795). Importantly, the molecular function collagen binding (Supplemental Table S1*C*; https://doi.org/10.5281/zenodo.3532795) was enriched in both populations upon bleomycin treatment, suggesting that both *Acta2-tdT* and *Pdgfra-tdT* cells are associated with collagen.

### Murine Models Show Cellular Collagen Expression with Both αSMA- and PDGFRα-expressing Cells but Preferential Association of Desmin with αSMA-expressing Cells

Our human histological analysis and RNA-Seq results revealed shared collagen production, but skewed expression of contractile machinery proteins between αSMA- and PDGFRα-expressing cells. In the final step, we therefore investigated the association of collagen and desmin marker expression with each of those cell populations in murine pulmonary fibrosis models.

**Fig. 8.**
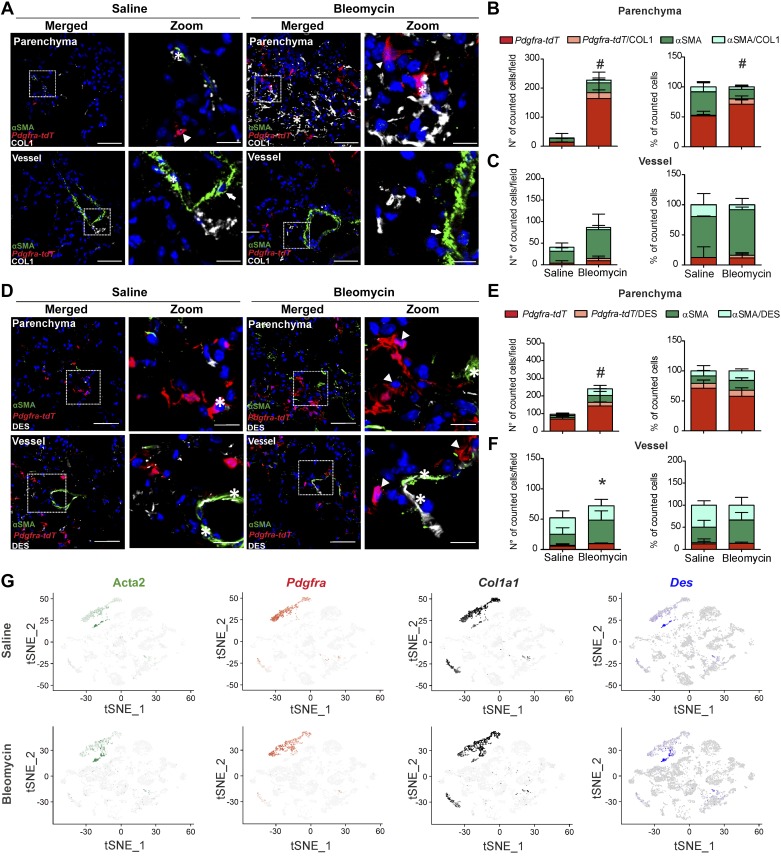
A fraction of both α-smooth muscle actin (αSMA) and platelet-derived growth factor receptor-α (PDGFRα)-positive cells express collagen 1 (COL1), while desmin (DES) is expressed mostly by αSMA-expressing cells in bleomycin. *A*: lineage tracing of PDGFRα-positive cells (*Pdgfra-tdT*) and immunofluorescence staining for αSMA and COL1 in saline- and bleomycin-treated mice. Parenchymal and vascular regions are shown. Scale bar: single staining and merged 50 µm; zoomed merged 12.5 µm. Arrows: single αSMA-positive cells; arrowheads: single *Pdgfra-tdT*-positive cells; asterisk: double αSMA/COL1 or *Pdgfra-tdT*/COL1-positive cells. Number and percentage of single αSMA, single *Pdgfra-tdT*, αSMA/COL1 and *Pdgfra-tdT* /COL1 double positive cells in the parenchyma (*B*) and in the vasculature (*C*). *P* value < 0.05 was considered significant. #Differences between single *Pdgfra-tdT* positive cells in bleomycin compared with saline. *D*: lineage tracing of PDGFRα-positive cells (*Pdgfra-tdT*) and immunofluorescence staining for αSMA and DES in saline- and bleomycin-treated mice. Parenchymal and vascular region are shown. Scale bar: merged 50 µm; zoomed merged 12.5 µm. Arrows: single αSMA-positive cells; arrowheads: single PDGFRα-positive cells; asterisk: double αSMA/DES or PDGFRα/DES-positive cells. Number and percentage of αSMA, *Pdgfra-tdT*, αSMA/DES, and *Pdgfra-tdT* /DES double positive cells in the parenchyma (*E*) and in the vasculature (*F*). *P* value < 0.05 was considered significant. #Differences between single *Pdgfra-tdT*-positive cells; *differences between single αSMA-positive cells in bleomycin compared with saline treatment. *G*: *t*-distributed stochastic neighbor embedding (tSNE) plots showing expression of Acta2, Pdgfra, Col1a1, and Des on the fibroblasts cluster upon saline and bleomycin treatment. Data from the Peyser et al. data set (GSE129605) (23).

In contrast to the human lungs where nearly all PDGFRα*+* cells were positive for collagen, in the bleomycin model only ~10% of parenchymal and ~30% of perivascular *Pdgfra-tdT*+ cells costained with COL1 (Fig. 8, *A*–*C*). Moreover, bleomycin treatment showed ~20% of parenchymal and ~10% of perivascular double αSMA/COL1 + cells (Fig. 8, *B* and *C*)

Similar to the human lungs, DES expression was observed preferentially in αSMA+ cells in bleomycin (Fig. 8*D*). Interestingly, while we observed an ~20–30% increase in αSMA+/DES+ cells in the parenchyma of bleomycin, the percentage of double positive cells decreased by ~30% in the vascular compartment of bleomycin compared with the saline controls (Fig. 8, *E* and *F*). These finding were further substantiated by analyzing an independent public scRNA-Seq data set (GSE129605) derived from lungs of saline- and bleomycin-treated mice (23). In the fibroblast cell pool of both bleomycin- and saline-treated mice, the expression of Acta2 and Pdgfra was limited to distinct cell subsets with limited overlap (Fig. 8*G*). Similar to the fluorescence approach, expression of collagen (Col1a1) expression was detected in both Acta2 and Pdgfra subsets, while desmin (Des) was mostly restricted to Acta2 cells (Fig. 8*G*).

**Fig. 9.**
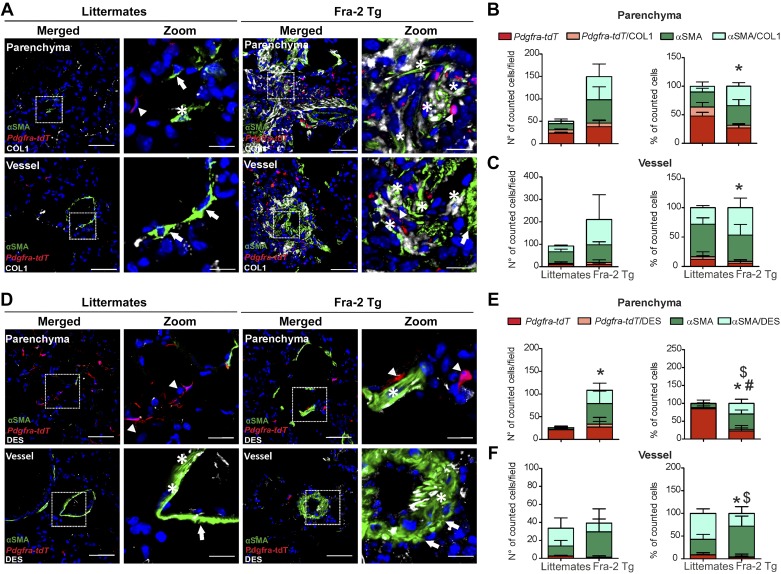
A fraction of both α-smooth muscle actin (αSMA)- and platelet-derived growth factor receptor-α (PDGFRα)-positive cells express collagen 1 (COL1), while desmin (DES) is expressed mostly by αSMA-expressing cells in *Fra-2 Tg* mice. *A*: lineage tracing of PDGFRα-positive cells (*Pdgfra-tdT*) and immunofluorescence staining for αSMA and COL1 in wild-type (wt) littermates and *Fra-2 Tg* mice. Parenchymal and vascular regions are shown. Scale bar: merged 50 µm; zoomed merged 12.5 µm. Arrows: single αSMA-positive cells; arrowheads: single PDGFRα-positive cells; asterisk: double αSMA/COL1 or PDGFRα/COL1-positive cells. Number and percentage of single αSMA, single *Pdgfra-tdT*, αSMA/COL1, and *Pdgfra-tdT*/COL1 double positive cells in the parenchyma (*B*) and in the vasculature (*C*). *P* value < 0.05 was considered significant. *Differences between single αSMA-positive cells in *Fra-2 Tg* mice compared with wt littermates. Cell count was performed in 3–5 images for mouse section from *n* = 2–3 different littermate controls and *Fra-2 Tg* mice. *D*: lineage tracing of PDGFRα cells (*Pdgfra-tdT*) and immunofluorescence staining for αSMA and DES in wt littermates and *Fra-2 Tg* mice. Parenchymal and vascular regions are shown. Scale bar: merged 50 µm; zoomed merged 12.5 µm. Arrows: single αSMA-positive cells; arrowheads: single *Pdgfra-tdT*-positive cells; asterisk: double αSMA/DES or *Pdgfra-tdT*/DES-positive cells. Number and percentage of αSMA, *Pdgfra-tdT* single positive, αSMA/DES, and *Pdgfra-tdT*/DES double positive cells in the parenchyma (*E*) and in the vasculature (*F*). *P* value < 0.05 was considered significant. #Differences between single *Pdgfra-tdT*-positive cells; *differences between single αSMA-positive cells; $difference between double αSMA/DES-positive cells in *Fra-2 Tg* mice compared with wt littermates. Cell count was performed in 3–6 images for mouse section. *n* = 3–4 different littermate controls and *Fra-2 Tg* mice.

In *Fra-2 Tg* mice, collagen deposition in the vascular compartment was more pronounced than in bleomycin-treated mice and was observed both peri- and intravascularly (Fig. 9). Similar to the bleomycin model, only ~10–20% of parenchymal and ~30–40% of perivascular *Pdgfra-tdT*+ cells costained with COL1 (Fig. 9, *B* and *C*) in *Fra-2 Tg*. The percentage of double αSMA+/COL1+ cells was, similar to the human lungs, ~50% and ~65% of in the parenchymal and vascular compartments, respectively (Fig. 9, *B* and *C*). DES expression was again almost exclusively observed in αSMA+ cells (Fig. 9, *D*–*F*).

## DISCUSSION

Understanding the cellular heterogeneity accompanying parenchymal and vascular remodeling is an important step in the development of novel antifibrotic therapies. Previously, myofibroblasts (αSMA+ cells) were speculated to be the main source of collagen production in the lung, however, this paradigm has recently been questioned in mouse lung fibrosis (29). Furthermore, recent studies have given first insights into underlying differences in mesenchymal populations (25, 34). It is becoming increasingly obvious that the mesenchymal cell pool is a highly heterogeneous mixture of cells, yet how much of this difference is due to predetermined cell fates and different lineage trajectories versus more plastic cell states and phenotypic modulation is currently unknown. The main finding of our study is that transcriptionally distinct cell populations marked by high expression of either αSMA or PDGFRα represent independent lineages in adult lungs with a characteristic contribution to parenchymal and vascular remodeling that is shared between murine models and human patients.

Using a binary lineage-tracing approach (*Acta2-tdT and Pdgfra-tdT*) in two distinct animal models of lung fibrosis (bleomycin and *Fra-2 Tg*), we show anatomically preferential localization of αSMA- and PDGFRα-expressing cells at baseline and in fibrosis. We identified a compartment-specific fibrotic response in which vascular remodeling is mostly represented by the expansion of resident αSMA+ cells, with the contribution of PDGFRα+ cells limited to the perivascular regions, and parenchymal remodeling consisting predominantly of PDGFRα+ cells. Importantly, the fate-mapping approach supported the immunofluorescent histological analysis showing limited overlap between αSMA and PDGFRα expression. The relatively low number of lineage-labeled cells that are coexpressing both αSMA and PDGFRα indicate a low transdifferentiation potential between these two cell populations in adult lungs and imply that even during a fibrotic response, these two populations are maintained and behave as separate lineages. This data interpretation was further supported by the analysis of open access scRNA-Seq data set from human donor and IPF lungs deposited in the public domain by an independent research group (25). Additionally, our global gene expression analysis revealing divergent transcriptome profiles between the two populations further suggest not only separate lineages but specialized biological roles as well. Our results suggest that independent accumulation of these cell types is the main cellular mechanism underlying pathological lung remodeling in adulthood. This is in contrast to a recent study that used a tetracycline-inducible Pdgfra lineage tracer line and reported that up to 40% of myofibroblasts originate from Pdgfra lineage upon bleomycin injury (17). The reason for this discrepancy with our results is currently unknown and would require additional clarification but could be the result of efficiency and specificity of tamoxifen labeling and use of different myofibroblast markers (αSMA versus SM22α). Different lineage tracing mouse lines and strain could also affect the results as we have observed a lower number of labeled PDGFRα between saline and wild type littermate controls.

The observed differences between these two major populations on a cellular level could form a basis of variable disease progression and heterogeneity observed in the clinical setting. Indeed, a growing body of evidence indicates that diverse lung-resident mesenchymal populations contribute to pulmonary fibrosis (27, 32, 34). This is particularly mirrored by their specific set of genes upregulated in fibrotic condition. While αSMA + cells upregulated mostly ECM genes, PDGFRα+ cells were mostly enriched in inflammatory and ribosomal genes.

The involvement of vascular remodeling in fibrotic lung disease is an underinvestigated field. We have previously shown extensive vascular remodeling in advanced IPF and specific gene expression patterns (11). However, cellular changes in the vascular compartment and the fate of different cell types during fibrosis was not addressed. In this study, we have demonstrated that vascular αSMA+ cells originate from pre-existing smooth muscle cells applying two independent lung fibrosis models. This is further supported by the exclusive expression of the contractile marker DES on αSMA+ cells. This is in line with our previous study where similar results were obtained by using different remodeling stimuli such as hypoxia or allergen challenge (7, 19). Additionally, in both our murine models we could show that the percentage of αSMA+ cells expressing DES decreased, while those positive for COL1 increased, suggesting either a shift from a contractile to a synthetic phenotype in αSMA+ cells or expansion of the COL1+/αSMA+ population in fibrotic conditions. In human IPF, COL1- and DES-positive αSMA+ cells were both increased, suggesting a different dynamic of αSMA-mediated remodeling in mice and humans. It is also possible that the observed differences reflect different stages of the remodeling process, which is still ongoing in mice but at an end stage in the human IPF samples.

The contribution of αSMA+ cells in pulmonary fibrosis has been extensively addressed; however, the source of parenchymal myofibroblasts remains elusive. A recent study reported axis inhibition protein 2 (Axin2)+ cells as the source of αSMA-expressing myofibroblasts upon injury (35), while double Axin2/PDGFRα-positive cells represented a distinct lineage showing a proliferative response upon lung injury (35). Another study reported that glioma-associated oncogene homolog 1 (Gli1)-positive cells give rise to αSMA+ cells, while only a minor percentage of Gli1+ cells retained PDGFRα expression in the lung (14). However, an independent study delineating the transcriptional consequences of hedgehog pathway activation in pulmonary fibroblasts revealed major effects on cell cycle genes, but no increase in contractile machinery, indicating a lack of transdifferentiation into myofibroblasts (22). Similarly, we demonstrate an expansion of PDGFRα+ cells with minimal αSMA coexpression.

This is in contrast to observations from lung development studies where PDGFRα+ cells have been identified as the main source of interstitial αSMA+ cells (alveolar myofibroblasts) (21), implying the importance of contextualizing the obtained finding. Possible explanations for these differences could be the loss of cell plasticity of a common progenitor cell in adulthood. While PDGFRα expression might be a shared intermediate stage among different lineages during development, cell marker expression and behavior could become restricted in adulthood. The small number of αSMA+/PDGFRα+ cells in both normal and fibrotic conditions could therefore indicate a special subpopulation of PDGFRα+ or αSMA+ cells. It remains an open question whether the source of these αSMA+/PDGFRα double positive cells is through acquisition of PDGFRα expression by some progenitors of the αSMA+ lineage, such as Adrp+ (10), Axin2+ (35), or Tbx4+ cells (32), or alternatively acquisition of αSMA expression from a small subset of PDGFRα lineage cells. Another possibility is that the αSMA+/PDGFRα+ population is established early in development as a separate lineage that persists as a small cell population into adulthood. Irrespective of the origin of the double positive cells, our reciprocal fate mapping approach effectively excludes a significant contribution of this subpopulation to fibrotic remodeling. The overarching conclusion is that the appearance and expansion of parenchymal αSMA+ cells (myofibroblasts) seem to be independent of PDGFRα+ cells in adulthood, but rather controlled by different cellular mechanisms, possibly including expansion of resident alveolar myofibroblasts and upregulation of αSMA expression in other lineages.

Our results from lineage tracing and immunofluorescence staining in bleomycin and *Fra-2 Tg* animal models and in human IPF lungs revealed that both PDGFRα+ and αSMA+ cells exhibit collagen positivity. This finding was also confirmed in our bulk RNA-Seq data set and further supported by an independent data set derived from bleomycin- and saline-treated mice (23). The finding of collagen production being not exclusively produced by αSMA+ cells but also by PDGFRα+ cells is in line with a recent study showing that in bleomycin-induced lung fibrosis, PDGFRα+ cells are the main matrix producing fibroblasts while only a subset of αSMA+ cells expressed collagen (34). We confirmed and expanded on these findings of the two cellular populations with compartment specificity in two animal models and most importantly in human samples. While we observed an involvement of PDGFRα+ cells in collagen production during the active phase of lung fibrosis development, αSMA+ cells have been shown to play an important role in the resolution phase of bleomycin-induced lung fibrosis (10), suggesting that the contribution of these two cell populations to the lung fibrosis process also depends on their different temporal appearance.

A key conclusion from the present study is that the biology not only of αSMA+ cells, but also that of PDGFRα+ cells should be incorporated into studies focusing on lung fibrosis. Future studies aimed deciphering the underlying molecular pathways governing the expansion and appearance of distinct fibroblast populations could offer novel and more efficient therapeutic targets for parenchymal and vascular remodeling.

## GRANTS

V. Biasin is supported by the Austrian Science Foundation (FWF, T1032-B34). The study was funded by the Jubilee Foundation of the Austrian National Bank (grant 16187 to G. Kwapiszewska) and the Austrian Science Foundation (FWF, grant P 27848-B28). S. Bellusci acknowledges the funding of the Deutsche Forschungsgemeinschaft (CRC1213, project A04), and S. Crnkovic was supported by a European Respiratory Society Long Term Fellowship (LTRF201801-00308).

## DISCLOSURES

No conflicts of interest, financial or otherwise, are declared by the authors.

## AUTHOR CONTRIBUTIONS

V.B., S.C., A.B., and G.K. conceived and designed research; V.B., S.C., A.S.-O., A.B., K.S., and W.K. performed experiments; V.B., A.S.-O., A.B., and L.M.M. analyzed data; V.B., S.C., and G.K. interpreted results of experiments; V.B., A.B., and S.B. prepared figures; V.B., S.C., and A.S.-O. drafted manuscript; V.B., S.C., A.B., E.E.A., S.B., L.M.M., and G.K. edited and revised manuscript; V.B., S.C., A.S.-O., A.B., E.E.A., K.S., W.K., A.O., S.B., L.M.M., and G.K. approved final version of manuscript.
